# Evaluating the Efficacy of Government Spending on Air Pollution Control: A Case Study from Beijing

**DOI:** 10.3390/ijerph16010045

**Published:** 2018-12-25

**Authors:** Xiaoyao Xie, Yuhong Wang

**Affiliations:** 1 School of Law, Ningbo University, Ningbo 315211, China; xiexiaoyao@nbu.edu.cn; 2 School of Business, Jiangnan University, Wuxi 214122, China

**Keywords:** government financial input, ambient air quality, environment, pollution

## Abstract

The reform and opening up of the Chinese economy over the last 40 years has led to rapid economic development. However, with the rapid expansion of the economy, increasingly serious air pollution is apparent. In order to control urban air pollution effectively, Chinese governments at all levels have invested large sums every year. However, it has become a difficult issue which influences public government decisions with respect to how and according to what standard to distribute financial funds so as to improve air quality while saving money at the same time. Taking Beijing as an example, this paper investigates the ten-year change in the annual daily mean of inhalable particulate matter (PM_10_), sulfur dioxide (SO_2_), and nitrogen dioxide (NO_2_) from the year of 2006 to 2015, researches the invested funds in environmental protection in Beijing, and establishes a relationship between the atmospheric indexes of the above three parameters and government-invested funds in environmental protection. According to model analysis, government financial input has an obvious influence on the improvement of air quality. However, during the long period of financial input, the degree of air quality improvement will reduce gradually as time goes by. There exists a direct link between the effectiveness of government financial input to promote air quality and the air quality index, which means when the pollutant standards index is poor (i.e., the corresponding pollutant concentration is higher), the effectiveness will be more apparent. On the contrary, when the index is at a good level, the effectiveness of government financial input is very small. To achieve the best air quality conditions, the government should set the detailed financial input at or over the first-grade standard according to urban air quality standards.

## 1. Introduction 

The Chinese economy has undergone rapid development because of reform and liberalization over almost 40 years, which greatly satisfies people in terms of income, education, and other aspects. Nevertheless, more and more serious environmental problems have appeared as a result of the rapid expansion in the Chinese economy. According to the “Environmental Performance Index” report in 2014, China ranked the 118th out of 178 countries. As to the rank of air quality, China ranked the second worst, with a score of 18.81, a decrease of 14.15% compared with ten years ago. In the light of the World Health Organization (WHO) investigation report on global urban air quality in 2014, only nine Chinese cities entered the list of the first 100 cities reaching the air quality standard. The deterioration of China’s environmental quality has become so serious that it influences the residents’ health. It is time to solve environmental problems and heighten the level of ecological civilization construction.

Ecological civilization construction is one of the most important contents of the nation’s governing modernization, and finance is the basis as well as the crucial support of the nation’s governing. Hence, finance plays a critical role in ecological civilization construction. From the year 2007 to 2014, China’s financial expenditure in environmental protection increased by 3.78 times from 99.285 billion China Yuan to 375.224 billion China Yuan, with an average annual growth rate of 47.24%, far higher than the increase of financial expenditure and economic increase in the same year [1]. Thus, what is the environmental effect of the fast-growing public expenditure for the environment? It is indispensable to perform a quantitative analysis on the influence of environmental fiscal policy on the environmental treatment. As we know, researchers including Shen [2], Bao et al. [3], and Halkos et al. [4], etc. all contend that environmental fiscal policy had an apparent effect on the environmental treatment. The ensuing problem is that in what detailed sense the government taxation policy has an effect on the improvement of urban air quality. At present, China’s governments at all levels, especially provincial governments, have difficulty in making science-based public decisions to improve air quality. We consider whether it is possible to construct a comparably clear model which manifests the leverage effect of government financial input so that government can make the utmost of financial funds to improve urban air quality in the meantime, and satisfy the appeal for beautiful surroundings.

## 2. Literature Review

From the historical progress of many developed countries, with the development of economy, the relationship between environmental quality and economic evolution has shown an inverted U-shaped correlation, which means during the process of economic development, the environmental quality will become worse first and then get better [5]. Many countries and regions have paid a great price in terms of the environment during the process of economic development. In the period of treating environment pollution or maintaining good environment quality, the most common policy is to provide financial subsidies in order to restrict the pollutant emissions of those polluting enterprises and keep the economy developing steadily. According to the estimation of Coady et al. about 4.9 trillion US Dollars were used in 2013 as subsidies for fossil fuels all over the world; the subsidies increased to 5.3 trillion US Dollars in 2015 [6]. China provides large financial subsidies (up to 1.8 trillion US Dollars in 2013). Large financial subsidies will have a great impact on environmental quality, economic development, and so on. The degree of financial subsidies’ effect on the environment and how to regard financial subsidies are aspects that have attracted many scholars’ attention. To conclude, present research mainly embodies the following four aspects.

Firstly, some scholars analyze the relationship between government financial subsidies and the reduction of pollution discharge in the enterprises (especially those who pollute energy heavily) and explore their internal relations. Koplow held that financial subsidies have an influence on the price of energy technology, the use pattern, and investment of energy, pollution discharge, and so on [7]. Thus, carrying out a proper subsidy policy is beneficial to the improvement of environment quality. In the research of Chau et al., the authors affirmed the positive effects of financial subsidies and put forward a model to monitor pollution degree and subsidy degree. However, the financial subsidies for environment are also questioned [8]. Moltke thought that many forms of subsidies such as tax treatment, grants, and soft loans could even promote the production of traditional fossil fuels and the emission of pollutants, which would damage the environment to a large extent. Reform of financial subsidies is extremely necessary for the sustainable development of social economy [9]. Sohaili researched government departments’ subsidies for electricity, finding that this way of subsidies not only increases the usage of electricity but also extends the pollution of environment [10]. Owing to the fact that the subsidies for fossil fuels would contribute to the increase of energy consumption, Presley and other researchers probed deep into the consequences of Ghana removing subsidies for oil by means of constructing a multi-regional general equilibrium model. The results of the research showed that life welfare and economic development will also be affected while improving the environment. Before providing subsidies for oil, the government should assess the result in order to achieve a good balance by combining economic development and environment problems [11]. 

Secondly, the relation between government finance and the development as well as the exploitation of environmentally-friendly new energy is also the focus of some research. Many countries strongly advocate developing and using new energy in order to conserve energy and reduce emissions. For example, as a form of new and renewable energy, bioenergy takes full advantage of the straw of plants (e.g., crops). Not only can it supply energy, but it can reduce pollution, which is friendly to environment. The financial subsidies for bioenergy will put certain pressure on the government finance, but in some regions with a large area of crops the government financial subsidies are still worth a try on account of the great benefits the subsidy input brings. While reforming the way of traditional subsidies, government should reinforce the subsidies for renewable energy [12]. As far as China is concerned, reducing financial subsidies for fossil fuels and increasing that for renewable energy are means that will have a very limited influence on economic development and are of benefit to the improvement of environment quality on a macro basis. Emphasizing renewable energy and increasing subsidies for it will not only have a more everlasting influence on environmental benefits, but also have a continual and profound effect on the transition of energy structure, enhancement of energy use efficiency, and improvement of balancing energy distribution, etc [13]. 

Thirdly, there are researches which focus on particular industries and demonstrate the effectiveness of financial subsidy policy. By concentrating on the game-playing relationship between automobile users and government incentives, some scholars explored the feasibility between these two measures of raising fuel tax and subsidizing gas use to reduce the pollutant emission of motor vehicles, and investigated in what intervals these two measures can be effective to the utmost [14,15]. Other critics commented two situations of energy subsidy which are subsidies of the production end and consumption end, and their basis for use as well as advantages and disadvantages they may cause [16,17]. Qi contends that China should not only take its own conditions into account and the two measures above to subsidize, but should also have an emphasis in specific industries; according to the data analysis from 2006 to 2011, aiming at Compressed Natural Gas cars, the author concluded that the consumption end subsidy should be of primary importance at present [16]. Zhu adopted a top-down calculation method based on energy consumption to measure and calculate the carbon emission in China’s transportation from 1991 to 2011 [18]. Through applying a co-integration and error correction model, he performed an empirical analysis on influencing factors of carbon emission. The result shows that government must take effective measures to reduce the intensity of transportation energy, expand the use of CNG cars, and decrease the carbon emission in the turnover per unit. Xie et al. constructed a dynamic multi-stage game model to analyze the complicated dynamic relationship among consumers, taxi drivers, and government decision behavior [19]. By solving the model with the method of backward induction, they calculated the subsidy coefficient of unit production and carbon tax rate, both of which are ascertained by government, and the optimal carbon emission reduction rate, which is ensured by taxi operators. Xu and Wang discussed the relation between economic growth and logistic development and CO_2_ emissions [20,21].

Fourthly, some schloars expound and prove the distribution relation between ambient air quality and financial stimulus funds. Based on the distribution model and implementation mechanism of financial stimulus funds oriented by ambient air quality, Dong et al. did a case analysis on the urban PM monitoring data 2.5 from 2014 to 2015 and found that the model and mechanism which were scientific and operable could indeed provide management and technical support for the construction of the financial mechanism oriented by ambient air quality [22]. Xiong et al., by means of taking advantage of the provincial panel data from 2007 to 2013, examined environmental policies of China’s government to reveal whether under the local governments’ existing behavior the effect of fiscal policy and taxation policy on variables of air pollution treatment had threshold features based on per capita Gross Domestic Product (GDP), and to analyze the government competing transmission mechanism. Their findings are stated as follows. Firstly, local governments’ finance and tax policies had apparent threshold effects on the quality of regional air pollution treatment. Secondly, local environmental policy, which had great orientation, mainly concentrated on one or two pollution indicators. Thirdly, at present, neither fiscal policy nor tax policy had positive effectiveness to improve the quality of air pollution treatment. On the contrary, they had abnormal effects on fostering air pollution which were restrained with the development of economy. Fourthly, under the background of fiscal decentralization, government competition greatly impeded air pollution treatment [1]. Halkos and Paizanos used appropriate econometric methods to take the dynamic nature and the potential endogeneity in the relationships examined into account. As for SO_2_, there is estimated to be a direct negative effect incurred by government spending on per capital emissions, while the direct effect on CO_2_ pollution is insignificant. The indirect effect on CO_2_ spreads among the whole part of the sample range while the negative effect on SO_2_ only affects low-income levels and becomes positive as income increases. The overall effects appear subsequently to the patterns of the indirect effects which control their own direct ones for each pollutant [4]. From these results, government spending on income level in each considered country should be reasonably set in accordance with their own conditions. Li et al., using a multi-regional energy-environment-economy computable general equilibrium (CGE) model incorporating the direct abatement expenditure of the proposed policies, assess the impact of China’s air pollution abatement (APA) policies on both the economy and environment in the Beijing-Tianjin-Hebei (BTH) area, and arrive at one conclusion which end-of-pipe control is identified as the most cost-effective policy for most pollutant emission reductions, and that more joint measures are needed in future [23].

Existing research is exquisite and standard, but its defects are also clear. Firstly, the researchers laid stress on the amount of carbon emission in specific industries or fields, thus hoping to lower the standard or decrease emissions by virtue of government regulation means, like financial subsidies. In this way that the environment, especially air quality, could be improved. Secondly, some scholars recurred to exploring the differences of urban ambient air quality among different cities, constructing a distribution model and implementation mechanisms of financial stimulus funds to determine the ration of financial funds. However, these belated redeeming means could not provide predictive assistance to government public policy and could not satisfy people’s future expectation for required air quality. These problems, which reserve space for the further research, also become the key to solution. Taking Beijing as an example, the paper explores a ten-year change of the annual daily mean of inhalable particulate matter (PM_10_), SO_2_, and NO_2_ from the years 2006 to 2015, does research on the invested funds in the environmental protection in Beijing, and establishes a relation between the atmospheric indexes of the above three categories and government-invested funds in environmental protection. 

## 3. Model Construction

Although lacking overall and scientific evaluating indicators, environment quality as a broad conception still can be evaluated by indicators in single dimension like air, water environment, waste, forest environment, and so on [24]. In this model, we will set Beijing as an example and investigate a ten-year change of the annual daily mean of the inhalable particulate matter (PM_10_), sulfur dioxide (SO_2_), and nitrogen dioxide (NO_2_) from the year of 2006 to 2015, do research on the invested funds in the environmental protection in Beijing, and establish a relation between the atmospheric indexes of the above three categories and government-invested funds in environmental protection.

The data used to construct the model comes from Beijing’s statistical yearbooks of calendar year [25,26,27,28,29]. From the yearbooks, we cannot know in detail the invested funds of every environmental indicator in the total environmental protection invested funds, so before model construction, we suppose that the proportion of invested funds for the three atmospheric indexes in the total environmental invested funds is a constant. Namely,

(1) $$\left\{ \begin{array}{l} I_{PM}=\alpha_{PM}I_{all}\\ I_{SO_{2}}=\alpha_{SO_{2}}I_{all}\\ I_{NO_{2}}=\alpha_{NO_{2}}I_{all} \end{array}\right.$$

In Equation (1), *I_PM_*, *I_SO_2__*, and *I_NO_2__* represent invested funds separately for PM_10_, SO_2_, and NO_2_, respectively. *α_PM_*, *α_SO_2__*, and *α_NO_2__* are the proportions of corresponding invested funds in the total environmental invested funds, respectively. *I_all_* is the total environmental invested funds. Therefore, the equation relation between the improvement degree of air pollution indicators and total environmental input is as follows:

(2) $$Y_{im}=AI+\varepsilon$$

*Y_im_* here means the improvement degree of the environmental indicator. *I* represents invested funds. *A* is attenuation factor of invested funds, a function of *I*, and *ε* is estimated error. So, the relations of the three corresponding environmental indicators are as follows:

(3) $$\left\{ \begin{array}{l} Y_{imPM}=A_{PM}I_{PM}+\varepsilon_{PM}\\ Y_{imSO_{2}}=A_{SO_{2}}I_{SO_{2}}+\varepsilon_{SO_{2}}\\ Y_{imNO_{2}}=A_{NO_{2}}I_{NO_{2}}+\varepsilon_{NO_{2}} \end{array}\right.$$

In the model construction, we did research by mapping every invested fund to total invested funds for environmental protection and directly explored the correlation between the change of PM_10_ and SO_2_ as well as NO_2_ and total environmental invested funds.

Hence, the model relation between the improvement degree of air pollution indicators and total environment input is replaced as:

(4) $$Y_{im}=BI_{all}+\varepsilon$$

The model described in Equation (4) is called the model of financial input attenuation effect. In Equation (4), *B* is a function concerning *I*, covering the proportion of invested funds for every pollutant in total invested funds and the attenuation factor of invested funds.

According to Beijing’s statistical yearbooks, we know that the ten-year change of PM_10_, SO_2_, and NO_2_ from the years 2006 to 2015 and financial invested funds for environmental protection can be represented as shown in Table 1:

In Table 1, the means of PM_10_, SO_2_, and NO_2_ all take on a strong linear change with increased time. We can set the changes of three pollutants’ annual daily mean and years as a linear relation:

(5) P=bt+a

In the equation above, *P* is the pollutants’ annual daily mean, and *t* is the year as a time unit whose values are from the range of 2006 to 2015.

The estimated value of *b* and *a* can be obtained by the method of linear regression.

(6) $$\left\{ \begin{array}{l} \hat{b}=\frac{\sum_{i=1}^{n}\left(t_{i}-\bar{t})(P_{i}-\bar{P}\right)}{\sum_{i=1}^{n}{\left(t_{i}-\bar{t}\right)}^{2}}=\frac{\sum_{i=1}^{n}t_{i}P_{i}-n\bar{t}\bar{P}}{\sum_{i=1}^{n}t_{i}^{2}-n{\bar{t}}^{2}}\\ \hat{a}=\bar{P}-\hat{b}\bar{t} \end{array}\right.$$

By Equation (6), we can get the linear regressive parameters of PM_10_, SO_2_, and NO_2_:

(7) $$\left\{ \begin{array}{l} {\hat{a}}_{PM}=10.6986\\ {\hat{b}}_{PM}=-0.0053 \end{array}\right.$$

(8) $$\left\{ \begin{array}{l} {\hat{a}}_{SO_{2}}=7.2577\\ {\hat{b}}_{SO_{2}}=-0.0036 \end{array}\right.$$

(9) $$\left\{ \begin{array}{l} {\hat{a}}_{NO_{2}}=2.2128\\ {\hat{b}}_{NO_{2}}=-0.0011 \end{array}\right.$$

We can make fitting curves.

**Figure 1:**
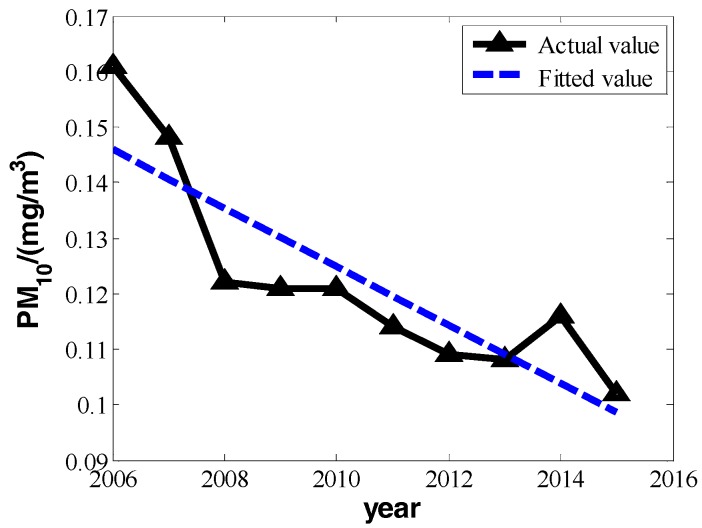
The change in the annual daily mean of inhalable particulate matter from 2006 to 2015.

**Figure 2:**
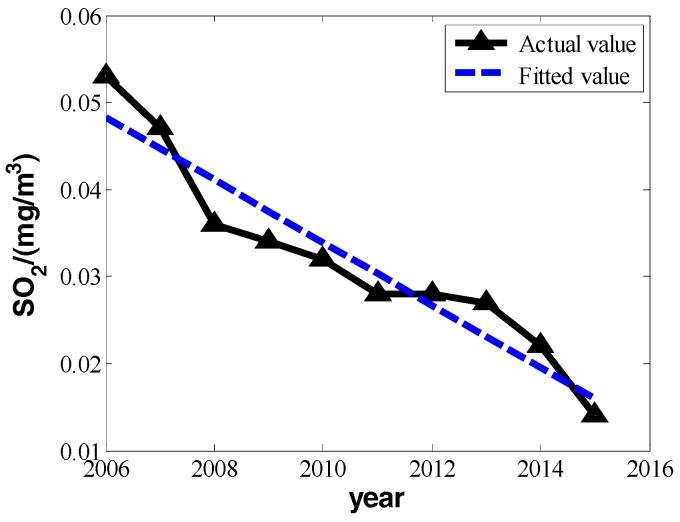
The change in the annual daily mean of sulfur dioxide from 2006 to 2015.

**Figure 3:**
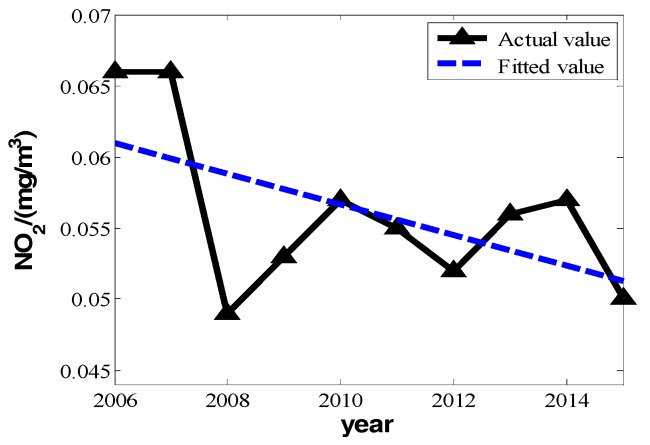
The change of nitrogen dioxide’s annual daily mean in 2006 to 2015.

From the three fitting curves of Figure 1, Figure 2 and Figure 3, we find the fitting efficiency on SO_2_ is the best and that on NO_2_ is the worst. On the whole, these three pollutants decrease linearly and the slope of decreasing tendency is $\hat{b}$, which actually means the degree of pollutant increase year by year. In the fitting curves, the value is a negative number. Combining the meaning of *Y_im_* in the model, $\left|\hat{b}\right|$ can be used as the measure of improvement degree. Namely, we consider that

(10) $$Y_{im}=\left|\hat{b}\right|$$

The model can also be:

(11) $$\left|\hat{b}\right|=BI_{all}+\varepsilon$$

In the equation above, the invested fund for environmental protection *I_all_* is not a value changing linearly, but it increases exponentially. In order to fit a growth curve, we can make a log transformation. The regression function is:

(12) $$\ln{\hat{I}}_{all}={\hat{b}}_{inv}t+{\hat{a}}_{inv}$$

We obtain the values of ${\hat{b}}_{inv}$ and ${\hat{a}}_{inv}$ by using Equation (6) with the method of linear regression:

(13) $$\left\{ \begin{array}{l} {\hat{b}}_{inv}=0.2896\\ {\hat{a}}_{inv}=-573.2396 \end{array}\right.$$

**Figure 4:**
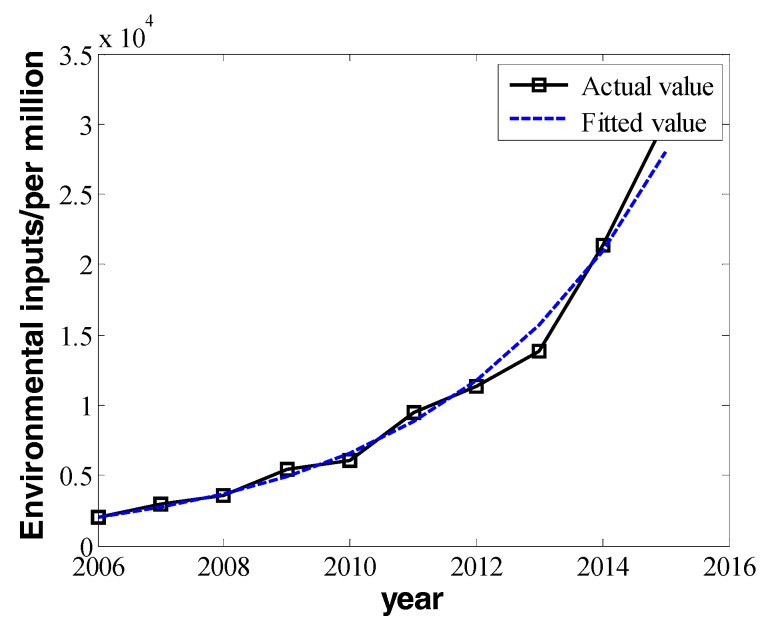
The change in invested funds for environmental improvement from 2006 to 2015.

Then, ${\hat{I}}_{all}=e^{{\hat{b}}_{inv}t+{\hat{a}}_{inv}}=e^{0.2896t-573.2396}$. We draw the fitting curve:

From Figure 4, we know the fitting degree of environmental financial invested funds is quite high.

In order to evaluate function *B*, the Equation (11) can be transformed simply:

(14) $$B=\frac{\left|\hat{b}\right|}{I_{all}}-\frac{\varepsilon }{I_{all}}=\frac{\left|\hat{b}\right|}{I_{all}}+\varepsilon^{\prime }$$

$\frac{\left|\hat{b}\right|}{I_{all}}$ here does not change linearly, but decreases exponentially. The detailed *B* values of PM_10_, SO_2_, and NO_2_ are:

(15) $$\left\{ \begin{array}{l} B_{PM}=\frac{\left|{\hat{b}}_{PM}\right|}{I_{all}}+\varepsilon^{\prime }=0.0053e^{-0.2896t+573.2396}\\ B_{SO_{2}}=\frac{\left|{\hat{b}}_{SO_{2}}\right|}{I_{all}}+\varepsilon^{\prime }=0.0036e^{-0.2896t+573.2396}\\ B_{NO_{2}}=\frac{\left|{\hat{b}}_{NO_{2}}\right|}{I_{all}}+\varepsilon^{\prime }=0.0011e^{-0.2896t+573.2396} \end{array}\right.$$

The above deduction only considers the impact of environmental investment for air quality in the current year, but as a matter of fact, the government’s environmental investment in previous years will also have a certain impact on the data of current year. Provided we take the previous years into consideration so as to make the model more rigorous, *Y*_(*t*)_ could be revised as follows:

(16) $$Y\left(t\right)=B_{0}\left(t\right)I_{all}\left(t\right)+B_{1}\left(t\right)I_{all}\left(t-1\right)+\cdots +B_{n}\left(t\right)I_{all}\left(t-n\right)$$

In Equation (16), $I_{all}\left(t\right)$ can be regarded as the government’s investment for environmental protection in year t, the degree of improvement brought by the investment before year *n* is $B_{n}\left(t\right)$. $I_{all}\left(t\right)$ changes exponentially, that is, $I_{all}\left(t\right)=kI_{all}\left(t-1\right)$. In addition, we assume that the impact of annual input on the next year is a fixed value *m*, that is $B_{n}\left(t\right)=mB_{n-1}\left(t\right)$. The previous conclusion shows that it is an exponential decay form, which can be assumed as $B_{0}\left(t\right)=We^{rt+h}$. Then Y(t) can be rewritten in Formula (17) as follows:

(17) $$\begin{array}{ll} Y\left(t\right) & =B_{0}\left(t\right)I_{all}\left(t\right)+kmB_{0}\left(t\right)I_{all}\left(t\right)+\cdots +k^{n}m^{n}B_{0}\left(t\right)I_{all}\left(t\right)\\ & =\left(1+km+\cdots +k^{n}m^{n}\right)B_{0}\left(t\right)I_{all}\left(t\right)\\ & =\left(1+km+\cdots +k^{n}m^{n}\right)We^{rt+h}I_{all}\left(t\right) \end{array}$$

Because the statistical data are quite small, and in order to extract the parameters conveniently, we only take the T year and T−1 year as example to extract the parameters. Taking the method of gradient descent to approximate the parameters imperatively, we find that W is very small for the three pollutants at last, and thereinafter we have the results of *W*.

(18) $$\left\{ \begin{array}{l} m_{NO_{2}}=1.48\times 10^{-7}\\ m_{PM10}=1.65\times 10^{-6}\\ m_{SO_{2}}=2.14\times 10^{-7} \end{array}\right.$$

As the result of Formula (18) states, the influent factor m of previous years is so small that it can be ignored reasonably. Hence, we just need to consider the investment of this year. The model of Formula (2) is applicable. 

In addition, two boundary conditions need to be added in order to prevent unreasonable data values, namely:

(1). *c_pollution_* > 0: The concentration of pollutants must be guaranteed to be greater than 0 and no negative values are given in data fitting.
(2). The fitting curve can be differentiated. The curves described in this paper are smooth and continuous curves which are necessary to investigate the rate of change in mathematical sense, so as to ensure that the curves are differentiated.

## 4. Model Analysis

This model starts with the change in environmental invested funds and the PM_10_, SO_2_, and NO_2_, and constructs the correlation among them. The *B* in the model function *Y_im_ = B × I_all_ +* *ε* is the key parameter to research on the effectiveness of environmental protection input. Its actual meaning is the amount of air quality improvement produced by the environment under present surrounding conditions with certain financial funds. From the final three results, we know with the government financial input for environmental protection year by year, the concentration or amount of every pollutant which measures air quality decreases year by year, but with time increasing, the improvement effectiveness reduces exponentially. If the time *t* is pushed back, with certain financial input the improvement effectiveness on air quality will reduce gradually and go to zero. That is to say, air quality will not improve at last, and the concentration of air pollutants will remain at a relatively stable value. 

The government’s input for environmental protection and the corresponding changes in the air quality improvement do share great similar with the marginal cost from the standpoint of economics. Generally speaking, the marginal cost increases first and then decreases with the change of production scale. There is indeed a certain degree of coincidence between the change of air quality improvement with government input and the change of marginal cost, to some extent, which reflect the law of marginal cost in the investment of environmental protection. 

In addition, from the process of model construction to parameter computation, we find the straight fitting degree of SO_2_ and PM_10_ is higher, and that of NO_2_ is lower. In order to probe deep into the source of the difference, we take the assessment of environment ambient air quality into consideration. China has administered a new air quality classification standard, GB3095-2012 [30], since 1 January 2016. The classification of inhalable particulate matter (PM_10_), SO_2_, and NO_2_ is shown in Table 2.

According to the data in Table 1, from 2005 to 2015, the concentration of inhalable particulate matter (PM_10_) reduced from 0.161 mg/m^3^ to 0.102 mg/m^3^. It improved to secondary standard from the level far below the standard of the three, and the concentration of PM_10_ had a decreasing tendency. The concentration of SO_2_ reduced from 0.053 mg/m^3^ to 0.014 mg/m^3^, improving from secondary standard to near first-grade standard. However, the change of NO_2_ is different. Although its concentration was comparably high in 2006 and 2007, it varied in other years in the range of 0.05 to 0.06 mg/m^3^, with a slight rangeability up and down the primary and secondary standards. Comparatively speaking, the concentration value of NO_2_ is small, and so is its corresponding improving space. From 2008 to 2015, the average decrements of NO_2_ were almost zero, which indicated that the input for environmental improvement had a very small or even no effect on later NO_2_ improvement. Comparing the variation amount of PM_10_, SO_2_, and NO_2_, for the item with the worse quality index, the improvement effectiveness of environmental financial input is larger, but for the item with better index, the effectiveness is very limited or even approaches zero. However we cannot obtain the distinct relativity from the fitting curve of NO_2_ compared with that of PM_10_ and SO_2_. However, from another perspective, it indicates that as time goes by the effectiveness of environmental financial input will gradually decrease with the improvement of corresponding index.

From the analysis above, we can get the following conclusions:

(1). The improvement of government financial input for air quality is apparent, but during the long-term financial input, the improvement degree of air quality will gradually reduce and eventually disappear with time going by.
(2). There is a direct relevance between the improvement effectiveness of government financial input for air quality and indicators of air quality. That is to say, if the air pollution indicator is worse (the corresponding pollutant concentration is bigger), the improvement effectiveness will be more distinct. On the contrary, if the air pollution indicator has been in a better level, then the environmental financial input will have quite small or even no improvement effectiveness.
(3). The effectiveness of government financial input to improve air quality is limited. In order to make the most of financial funds to the maximum, government should make a dynamic plan of financial input; in order to achieve optimal state of air quality, financial input should be set on or over the first-grade standard.

## 5. Prediction 

This paper establishes the relation between Beijing’s financial input for environmental protection and the change rate of PM_10_, SO_2_, and NO_2_, and uses the ten-year data from 2006 to 2015 to obtain a detailed parameter which can be used not only to analyze the performance financial input for the environmental protection has been achieved in the past ten years, but predict and simulate future input and its performance. Here, we make use of the constraint conditions in model function and model analysis, and predict the change of PM_10_, SO_2_, and NO_2_ in the next five years.

**Figure 5:**
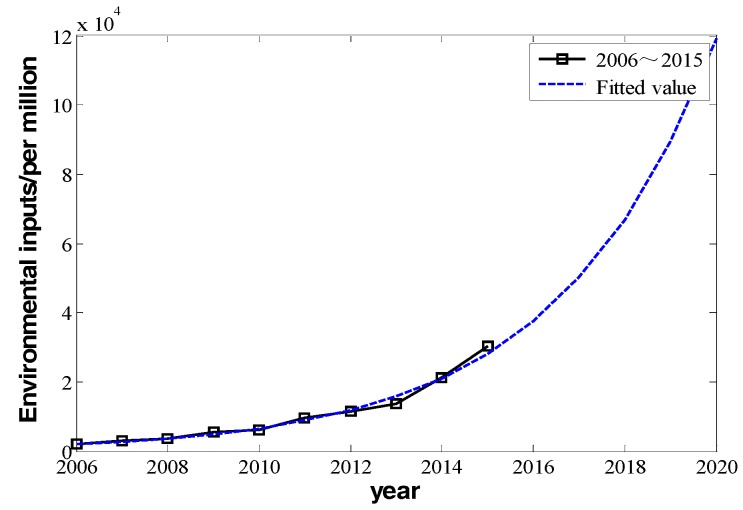
The actual value and predicted value of environmental input in 2006 to 2020.

Over the past ten years, Beijing has attached much more importance to environmental protection. The proportion of financial invested funds for environmental protection in GDP is larger and larger. According to the statistical yearbooks of Beijing, the proportion of financial input for environmental protection in Beijing’s GDP increased from 0.25% to 1.3% from 2006 to 2015, respectively [31,32]. The increasing speed is expected to be maintained in the next five years and increase to over 2.5% in 2020 [33]. We assume that Beijing’s GDP will maintain steady growth every year, and then Beijing’s invested funds for environment still keep increasing exponentially, which means $I_{all}=e^{0.2896t-573.2396}$. Then, by Equation (13), the prediction of environmental invested funds in the next years can be show in Figure 5.

### 5.1. Prediction of PM_10_

From the change value of PM_10_ in Table 1 and the air quality classification in Table 2, we know the concentration of PM_10_ gradually reduced in the decade from 2006 to 2015, but it did not reach the required secondary standard in Table 2 until 2015. It is still high and with large space for reduction. Then according to the model analysis, the model is completely suitable for the prediction of change in the next five years. Based on $I_{all}=e^{0.2896t-573.2396}$ and according to the model,

(19) $$\left\{ \begin{array}{l} Y_{PM\_pre}=B_{PM\_pre}I_{all}+\varepsilon \\ B_{PM\_pre}=k_{PM\_pre}e^{-0.2896t+573.2396} \end{array}\right.$$

**Figure 6:**
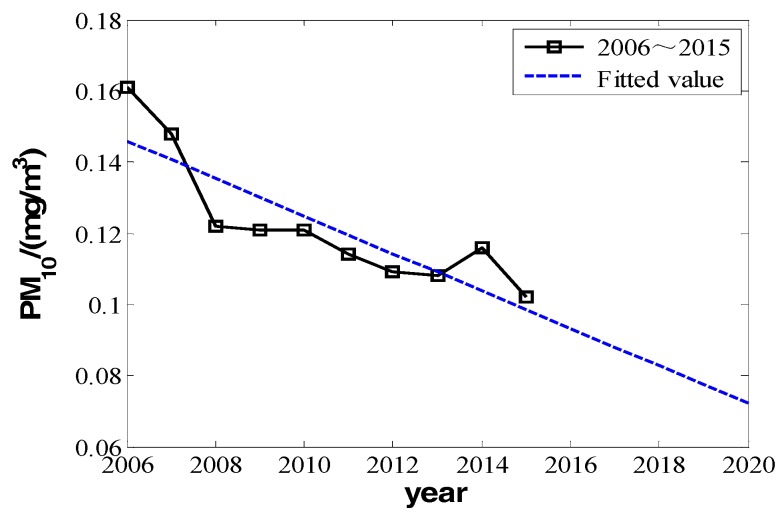
The actual value and predicted value of inhalable particulate matters’ annual daily mean in 2006 to 2020.

From Equation (19), $Y_{PM\_pre}=k_{PM\_pre}+\varepsilon$ is a constant, which means the change amount of PM_10_ is a constant, similar to that in Equation (7). Its change curve can be seen in Figure 6. From the predicted curve, we get the information that to 2020 the concentration of PM_10_ can reduce to about 0.07 mg/m^3^, reaching the required secondary standard in Table 2.

### 5.2. Prediction of NO_2_

**Figure 7:**
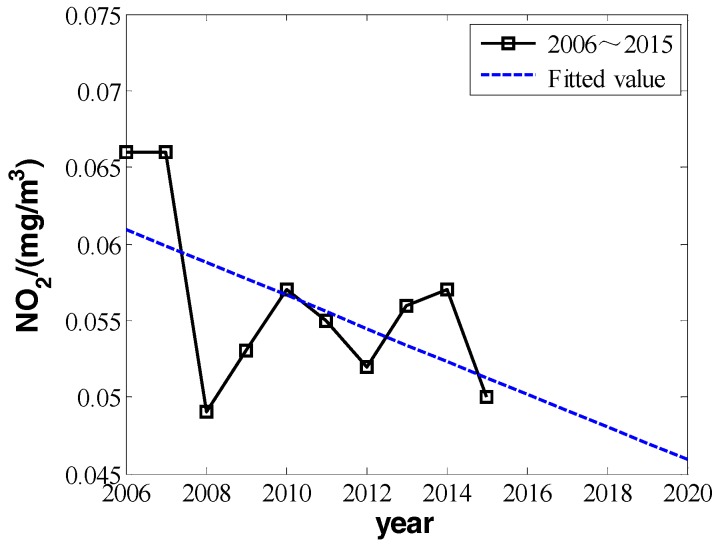
The actual value and predicted value of nitrogen dioxide’s annual daily mean in 2006 to 2020.

From Table 1 and Table 2, the annual daily mean of NO_2_ took on a decreasing overall tendency in the decade from 2006 to 2015, not reaching the required secondary standard in Table 2. Although it is with high relative volatility and its decreasing tendency is not as obvious as that of PM_10_, its overall change tendency still can be predicted by using model function. Similar to PM_10_, the change amount of NO_2_ is also a constant in the next five years, that is $Y_{NO_{2}\_pre}=k_{NO_{2}\_pre}+\varepsilon$. Its linear parameter decreasing linearly is the same as Equation (9), and its changing curve is shown in Figure 7. From the prediction in Figure 7, the annual daily mean of NO_2_ will reduce to around 0.045 mg/m^3^, which is quite close to secondary standard in Table 2.

The model has a good linear prediction effectiveness on PM_10_ and NO_2_, because their annual daily mean was still large in 2015. However, the prediction for the change of SO_2_ cannot be similar to that for PM_10_ and NO_2_, because according the data in Table 1 and Table 2, the annual daily mean of SO_2_ was lower than 20 mg/m^3^ in 2015, lower than the required first-grade standard. Seeing from the statistic results, we know the concentration of SO_2_ in Beijing has been at a low level. If government continues to raise financial input for environmental protection, the effectiveness achieved will be very limited. In other words, the linear decrements of SO_2_ will not go on in the next five years. However, based on the fact that attenuation factor *B* in Equation (4) of model function is an exponential one, and in order to predict the change of SO_2_ correctly in the next five years, we suppose that in the next five years attenuation factor *B* will be larger. According to the model function, the degree of improvement *Y_im_*, is also the annual reduction amount of SO_2_’s annual daily mean. To conclude, it is an attenuating exponential function which is set as

(20) $$Y_{SO_{2}\_im}(n)=Ae^{-kn}$$

In Equation (20), $Y_{SO_{2}\_im}(n)$ is the variation of the year *n*. *A* and *K* are the function parameters. Then the annual daily mean of SO_2_ in the year *n + m* is

(21) $$\begin{array}{ll} V_{SO_{2}}(n+m) & =V_{SO_{2}}(n+m-1)-Y_{SO_{2}\_im}(n+m-1)\\ & =V_{SO_{2}}(n+m-1)-Ae^{-k(n+m-1)}\\ & =V_{SO_{2}}(n)-A\left[e^{-k(n+m-1)}+e^{-k(n+m-2)}+\ldots +e^{-kn}\right]\\ & =V_{SO_{2}}(n)-Ae^{-kn}\frac{1-e^{-km}}{1-e^{-k}} \end{array}$$

In Equation (21), *n* = 2015. In order to derive parameter *A* and *K*, we need to take in two boundary conditions. The first is $V_{SO_{2}}(n+m)\to 0$ when m→∞; the second is when *m* is equal to 0, the slope fitting $V_{SO_{2}}(n+m)$ is equal to the slope parameter in Equation (8).

(22) $$\left\{ \begin{array}{l} \underset{m\to \infty }{\lim}V_{SO_{2}}(n+m)=0\\ {\frac{d}{dm}V_{SO_{2}}(n+m)|}_{m=0}={\hat{b}}_{SO_{2}} \end{array}\right.$$

Taking the condition of Equation (22), we can derive the predicted expression of Equation (21) as,

(23) $$V_{SO_{2}}(n+m)=0.0159e^{-0.2256m}$$

**Figure 8:**
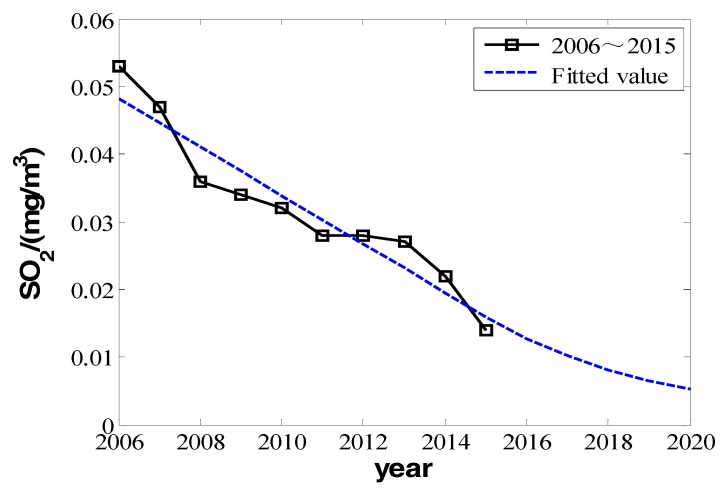
The actual value and predicted value of sulfur dioxide’ annual daily mean in 2006 to 2020.

As *n* here is equal to 2015, it can be taken into to derive the predicted value of SO_2_’s annual daily mean in future 5 years, which is shown in Figure 8. According to the predicted value, the annual daily mean of SO_2_ will reduce to 0.005 mg/m^3^ in 2020, which is far lower than the required first-grade standard in Table 2.

## 6. Conclusions 

Through the research on the degree of improving pollutant standards indexes by government financial input, we analyze the interior regulations between financial input and improvement effectiveness of air quality and find large part of financial input for environmental protection is invested as the financial subsidies for polluting enterprises [34]. This environmental input can achieve certain effectiveness on improvement or control against deterioration, so it is necessary to make a scientific plan of financial input and subsidies for environment. On the basis of this model, we draw the following conclusions.

(1) Financial subsidies play a critical role in the improvement of air quality, whose significance and effectiveness are extremely obvious, especially when air quality is in bad condition.
(2) There is no proportional relation between the input of government financial subsidies and the effectiveness of environmental improvement; when air quality is improved to certain standards, the effectiveness of financial input will reduce until to zero. Hence, we should take the input into overall consideration, combining the quota of government financial subsidies with expected effectiveness so as not to cause the waste of financial funds.
(3) In addition to government financial subsidies, we need to think of other factors to make air quality better continuously. For example, government departments should foster upgrade industries and enhance the emission reduction of the enterprises and sectors with high pollution and high emission [23,35]. Except for laws, regulations, and government policies, there are many other factors that should be taken into account as well as a pluralistic approach that should be to adopted in atmospheric governance [36]. For example, coordination and cooperation should be strengthened in various regions. It is necessary to carry out the united treatment between different regions. Besides, in the process of investigating the pollutant discharge, a comprehensive and balanced investigation of multiple pollutants is required rather than controlling nothing but a few pollutants in an extreme way [23]. Meanwhile, supervision departments should shoulder the responsibility of supervision and punish for illegal emissions according to the law. Government policymakers should positively foster and encourage the development of new energy and new technology with attention and preferential treatment.
(4) The country should define in the relevant laws and regulations for environmental protection the authority of local governments’ financial input and in the meantime enact the legal responsibility of governments’ not positively investigating financial input quota, which leads to financial waste.

## Figures and Tables

**Table 1:** Financial input for environmental protection and the changes of three air pollutants in Beijing from 2006 to 2015.

Year	Environmental Protection Input/million yuan	Annual Daily Mean of Inhalable Particulate Matter (mg/m^3^)	Annual Daily Mean of Sulfur Dioxide (mg/m^3^)	Annual Daily Mean of Nitrogen Dioxide (mg/m^3^)
2006	2013.65	0.161	0.053	0.066
2007	2927.28	0.148	0.047	0.066
2008	3546.88	0.122	0.036	0.049
2009	5404.59	0.121	0.034	0.053
2010	6085.41	0.121	0.032	0.057
2011	9451.35	0.114	0.028	0.055
2012	11,353.7	0.109	0.028	0.052
2013	13,816.72	0.108	0.027	0.056
2014	21,335.53	0.116	0.022	0.057
2015	30,326.12	0.102	0.014	0.050

**Table 2:** The table of air quality classification standards (unit mg/m^3^).

Pollutant	First-Grade Standard	Secondary Standard
PM_10_	0.04	0.07
SO_2_	0.02	0.06
NO_2_	0.04	0.04
